# Tracing theories in realist evaluations of large-scale health programmes in low- and middle-income countries: experience from Nigeria

**DOI:** 10.1093/heapol/czaa076

**Published:** 2020-09-03

**Authors:** Tolib Mirzoev, Enyi Etiaba, Bassey Ebenso, Benjamin Uzochukwu, Tim Ensor, Obinna Onwujekwe, Reinhard Huss, Nkoli Ezumah, Ana Manzano

**Affiliations:** 1 Nuffield Centre for International Health and Development, University of Leeds, Level 10 Worsley Building, Clarendon Way, LS2 9NL, Leeds, UK; 2 Health Policy Research Group, College of Medicine, University of Nigeria Enugu Campus, Old UNTH Road, Ogbete, Enugu State, Nigeria; 3 University of Leeds, School of Sociology & Social Policy, Social Sciences Building, Leeds LS2 9JT, UK

**Keywords:** Realist evaluation, programme theory, Nigeria, methodology

## Abstract

Realist evaluations (RE) are increasingly popular in assessing health programmes in low- and middle-income countries (LMICs). This article reflects on processes of gleaning, developing, testing, consolidating and refining two programme theories (PTs) from a longitudinal mixed-methods RE of a national maternal and child health programme in Nigeria. The two PTs, facility security and patient–provider trust, represent complex and diverse issues: trust is all encompassing although less tangible, while security is more visible. Neither PT was explicit in the original programme design but emerged from the data and was supported by substantive theories. For security, we used theories of fear of crime, which perceive security as progressing from structural, political and socio-economic factors. Some facilities with the support of communities erected fences, improved lighting and employed guards, which altogether contributed to reduced fear of crime from staff and patients and improved provision and uptake of health care. The social theories for the trust PT were progressively selected to disentangle trust-related micro, meso and macro factors from the deployment and training of staff and conditional cash transfers to women for service uptake. We used taxonomies of trust factors such as safety, benevolent concerns and capability. We used social capital theory to interpret the sustainability of ‘residual’ trust after the funding for the programme ceased. Our overarching lesson is that REs are important though time-consuming ways of generating context-specific implications for policy and practice within ever-changing contexts of health systems in LMICs. It is important to ensure that PTs are ‘pitched at the right level’ of abstraction. The resource-constrained context of LMICs with insufficient documentation poses challenges for the timely convergence of nuggets of evidence to inform PTs. A retroductive approach to REs requires iterative data collection and analysis against the literature, which require continuity, coherence and shared understanding of the analytical processes within collaborative REs.

**Key Messages**

- Realist evaluations (REs) are important, though time-consuming approaches to generating context-specific and practical implications for policy and practice within the ever-changing low- and middle-income countries (LMICs) health policy context. As such, REs reflect an applied nature of health policy and systems research.
- Theories in REs span across the continuum of middle-range theories—programme theories (PTs)—specific context, mechanism and outcome configurations. Realist researchers need to be cognizant of these levels of abstraction, including different interdependencies and overlaps between them, and need to ensure that PTs are ‘pitched at the right level’ to maintain methodological rigour alongside the policy relevance.
- Theory gleaning, development and testing require different nuggets of evidence. This is a challenge in REs, particularly in the context of LMICs where programme design and implementation are often insufficiently documented and researchers face difficulties in accessing relevant literature.
- The retroductive approach in REs requires continuous, iterative and flexible data collection and analysis against the current literature. Collaborative REs need to maintain the continuity, coherence and shared understanding of the analytical processes, though researchers also need to be cognizant of the need to maintain the momentum and focus within longitudinal studies.

## Introduction

Theory-driven evaluations, especially realist evaluations (REs), are increasingly popular in assessing health programmes and policies in low- and middle-income countries (LMICs) (McGaughey *et al.*, 2010; Prashanth *et al.*, 2012; Robert *et al.*, 2012; Vareilles *et al.*, 2015; Kwamie, 2016; Mirzoev *et al.*, 2016; Mbava and Rabie, 2018). REs involve iterative stages of theory gleaning, developing, testing, refining and consolidating from complementary ‘nuggets of evidence’ (Pawson and Tilley, 1997; Pawson and Manzano-Santaella, 2012; Manzano, 2016) including theoretical and empirical literature, programme documentation and implementation experiences. Substantial literature covers the underlying philosophy of REs (Marchal *et al.*, 2018), its aspects such as mechanisms (Dalkin *et al.*, 2015), processes of conducting REs (Emmel *et al.*, 2018) and quality standards (Wong *et al.*, 2016a). Despite their growing popularity (Prashanth *et al.*, 2012; Robert *et al.*, 2012; Vareilles *et al.*, 2015; Mirzoev *et al.*, 2016), methodological experiences of REs from LMICs are scarce (Gilmore *et al.*, 2019). Two specific challenges have been noted: how to frame programme theories (PTs) incorporating the concepts of contexts, mechanisms and outcomes (CMOs) and ‘positioning theory at the heart of the explanation’ (Marchal *et al.*, 2018). In REs, contexts comprise social, psychological, political, organizational, resources, historical and other aspects of programme setting or environment (Greenhalgh *et al.*, 2017d) and how and whether these aspects affect how particular mechanisms operate. Mechanisms are changes in reasoning and behaviour of individuals and are distinct from programme activities (Greenhalgh *et al.*, 2017c), opportunities and constraints. Outcomes refer to any observable patterns of changes due to programme implementation (Pawson and Tilley, 1997). Later, debates (Abimbola, 2019; Gilmore, 2019) also raised the role of (non)local researchers by highlighting power imbalances and risks of ‘limited contextual understanding or engagement and identifying appropriate PTs reflective of the context’ (Gilmore, 2019, p. 2).

REs are method-neutral and involve the retroductive analytical approach of moving between inductive and deductive reasoning to examine the causal powers of the policy or programme evaluated (Pawson and Tilley, 1997; Greenhalgh *et al.*, 2017a; Emmel *et al.*, 2018). Central to REs is the use of programme PTs as guides to explain how, why, for whom and in what circumstances programmes work (Pawson and Tilley, 1997; Mbava and Dahler-Larsen, 2019). Different models of PTs exist (Funnell and Rogers, 2011; Kneale *et al.*, 2015), all essentially involving descriptions ‘… in words or diagrams, of what is supposed to be done in a policy or programme (theory of action) and how and why that is expected to work (theory of change)’ (Greenhalgh *et al.*, 2017b). Researchers develop, test and refine PTs by examining causality, focusing on how contexts trigger specific mechanisms to produce intended or unintended outcomes - expressed as CMO configurations (Merton, 1968; Pawson and Tilley, 1997; Marchal *et al.*, 2018), or even sometimes adding intervention as a separate element resulting in ICMOs. Tested, refined and consolidated PTs inform wider middle-range theories, which are positioned at a higher level of theorization while still being close to observed patterns in the data (Merton, 1967; Greenhalgh *et al.*, 2017b). Thus, the ladder of abstraction in realist studies starts with specific findings, which inform testing and refinement of PTs through CMO configurations and eventually consolidation of middle-range theories (CDI, 2016).

In this article, we report and reflect upon experiences of ‘tracing’ (i.e. gleaning, developing, testing, consolidating and refining) two specific PTs - patient–provider trust and health facility security - within an RE of a national health programme in Nigeria. This article should be of relevance to researchers and practitioners who are interested in understanding specific theory-tracing processes within, and perhaps wider utility of, REs for policy and practice. Following a brief background of the programme, we document the processes of tracing each PT through identifying and comparing their analytical processes. We then discuss our experiences and outline key lessons learned.

### Background of the programme

During 2012–15, the Government of Nigeria implemented the Subsidy Reinvestment and Empowerment Programme (SURE-P), which aimed to support the social sector through reinvesting savings from the removal of fuel subsidies. One component focused on improving maternal and child health (MCH) outcomes (SURE-P/MCH), which was implemented in selected local government areas in the 36 states of Nigeria and in the Federal Capital Territory through clusters of four (4) Primary Health Care facilities plus one (1) general (secondary) referral hospital. SURE-P/MCH comprised supply and demand components. The supply component included recruitment and training of staff (2000 midwives, 10 000 community health workers), infrastructure development, improving availability of supplies and activation of Ward Development Committees, which through membership of local authorities and community leaders oversee performance of all public services including local health facilities. The demand component aimed to increase the utilization of MCH services through conditional cash transfers to pregnant women (The-Presidency, 2013; Mirzoev *et al.*, 2016).

## Methods

We report experiences from a longitudinal international collaborative RE of the SURE-P/MCH programme, conducted by the Universities of Leeds and Nigeria. Its distinctive feature included studying the sustainability of achieved changes following a sudden withdrawal of funding to the SURE-P/MCH in 2015. In practice, this meant that PTs looked beyond the funded implementation period, e.g. covering sustained improvement in the utilization of health care due to enhanced security and programme’s residual trust.

Our RE study, REVAMP (Dete***R***minants of ***E***ffectiveness and sustainability of a no***V***el community he***A***lth workers program***M***e in im*P*roving MCH in Nigeria), had three phases:

1. During phase 1 (June–October 2015), we developed a logic map of the SURE-P/MCH (Ebenso *et al.*, 2019) and gleaned initial working theories (IWTs) from programme documentation, literature on health systems strengthening and limited data collection.
2. During phase 2 (November 2015–September 2017), we developed PTs based on insights from more primary data alongside analysis of literature.
3. During phase 3 (September 2017–December 2019), we conducted several rounds of theory testing, refinement and consolidation through iterative data collection/analysis and literature review.

**Figure 1:**
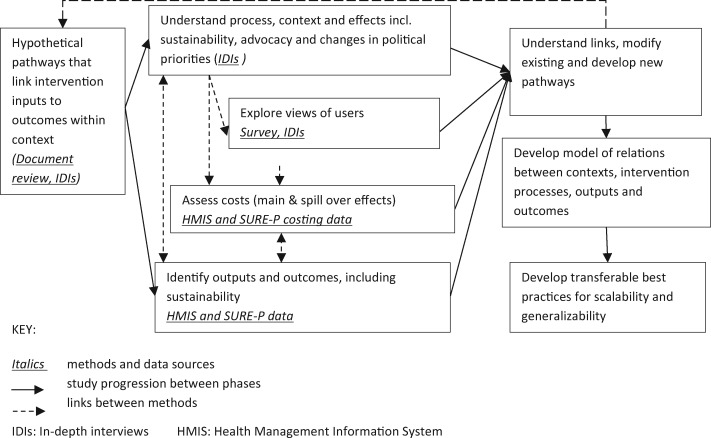
Study methods.

The study followed the RAMESES II quality and reporting standards and the recommended mixed-method approach for REs (Wong *et al.*, 2016a) shown in Figure 1, which combined quantitative [surveys, secondary analysis of data from the health management information system (HMIS)] and qualitative [document review, in-depth interviews (IDIs) and focus groups with key actors] methods. The study protocol is available elsewhere (Mirzoev *et al.*, 2016).

## Experiences of tracing realist theories

Our starting point was a broad theory of change summarized in the study protocol (Mirzoev *et al.*, 2016), which incorporated different elements of health systems strengthening (such as deployment of staff and provision of supplies) envisaged to improve equitable access to quality provision and improved the utilization of MCH services as the main outcomes of the SURE-P/MCH programme:

*Deployment of Community Health Workers, combined with health system interventions (e.g. infrastructure and supplies) and implemented within a favourable environment at individual, organisational and system levels, will improve: equitable access to quality maternal and child health services and ultimately the MCH health outcomes in Nigeria* (Mirzoev *et al.*, 2016, p. 5).

**Figure 2:**
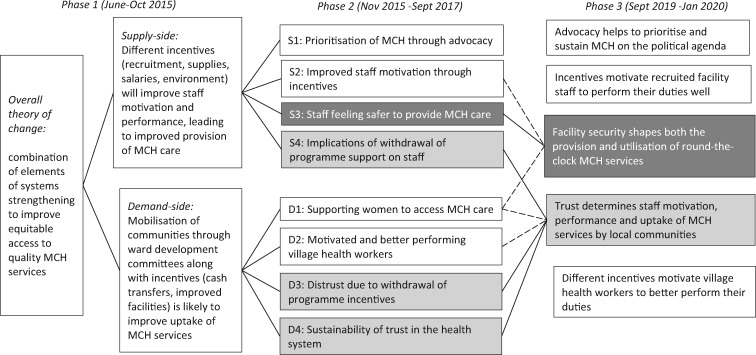
(1) Emergence of trust and security PTs throughout the study. Dashed lines show indirect links whereas solid lines show direct progression of theories and (2) darker grey shows emergence and progression of the security theory whereas lighter grey shows the same for the trust theory.

As shown in Figure 2, the overall theory was divided into two IWTs, which were subsequently sub-divided further into eight PTs (each with their own CMOs) and were eventually refined as our conceptualization became more nuanced throughout the project phases.

Our focus in this article on two specific PTs (trust and security) is based on two broad reasons. First, the patient–provider trust and health facility security PTs represent issues of complex and diverse nature. Trust is all encompassing, though it is not always a tangible phenomenon, whereas health facility security is more visible, and both go beyond the funded programme implementation period (2012–15), which relates to the sustainability of programme effects. Second, the importance of both trust and security emerged during the first phase, but each only became distinct PTs subsequently. This, combined with differences in their analytical trajectories, allowed us to compare and contrast our methodological experiences with tracing theories in REs throughout all three project phases.

### Phase 1. Theory gleaning: an overview of the programme and emerging key themes

During phase 1, we conducted preliminary discussions with programme architects, reviewed programme documentation, assessed data quality from the national HMIS and reviewed literature on health systems strengthening to improve MCH in LMICs. All these were discussed in-depth at a project meeting in the University of Leeds in September 2015. A major part of that meeting involved face-to-face capacity building on RE. This was followed by step-down capacity-building workshops at the University of Nigeria, facilitated by staff who attended the Leeds meeting for research staff who did not travel to that project meeting.

In the first version of our IWTs, we split our theory of change into the supply and demand parts, corresponding to the SURE-P/MCH programme logic (Ebenso *et al.*, 2019). Within each, we identified Cs, Ms and Os though did not articulate the causal links between them. As a consequence, the interview data were coded inductively and thematically to identify key themes.

Neither security nor trust was explicitly articulated in the SURE-P/MCH documentation, though they were implicit in the SURE-P manual’s references to improvements in working environment and infrastructure (security) and improved staff availability and training to enhance their performance (trust) (NPHCDA, 2012). Security was a more clearly evident theme from the primary data reflected in visible measures (such as facility perimeter fences or night guards), whereas trust emerged indirectly from themes raised by the programme architects and researchers such as health workers’ motivation (prerequisite for staff performance alongside training) and users’ confidence (prerequisite for improved use of health care referred to in the programme documentation).

Gleaning of PTs at this point was done through understanding the programme design and logic model, iterative discussions within research team and with programme architects and literature review as described in full elsewhere (Ebenso *et al.*, 2019). Our literature review mostly covered the empirical health systems literature, which focused on improving access to MCH services through health systems strengthening covering the supply side, i.e. provision of health services, and demand side, i.e. improving the uptake of health care (Agyepong *et al.*, 2014; Gopalan *et al.*, 2014). Even though at this stage we had relatively broad, and perhaps superficial, engagements with the literature on these two PTs, this was a necessary step to understand the programme architecture and logic, which helped surfacing the focus of our evaluation.

### Phase 2. Theory development: dealing with the theoretical magnifying glass

During phase 2, a more in-depth literature review was conducted, which informed data collection and analysis (96 IDIs, costing data, facility exit survey). Data analysis happened in five groups over 4 weeks, followed by a 2-day data harmonization and theory-building workshop in February 2017 in the University of Nigeria during which 15 themes were identified inductively (Box 1) and these themes then informed the development of 8 PTs.

**Figure 3:**
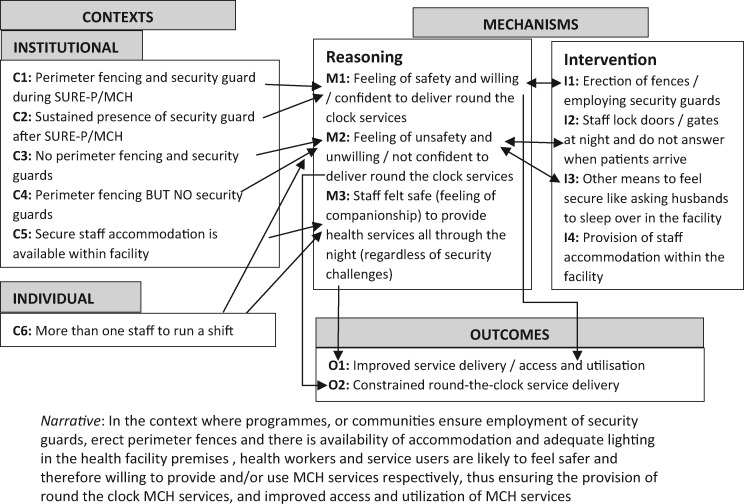
Visualization of security PT.

**Figure 4:**
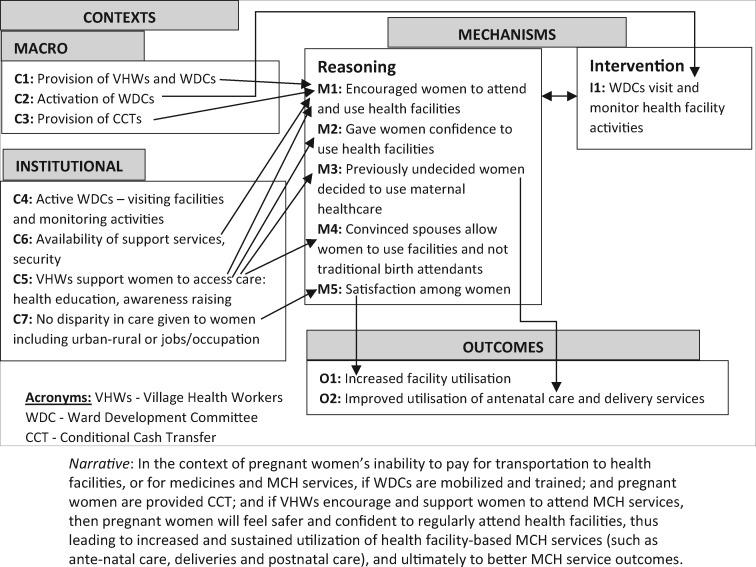
Visualization of trust PT.

Retroductive reasoning was used to develop PTs anchored in those 15 themes, which now were operationalized in tentative causality propositions. In subsequent months, these PTs were extensively peer-reviewed, contrasted with empirical and theoretical literature and informed iterative data collection and analysis. At this point, we included separate questions on trust and security in the data collection tools, and the interview data were coded by the Cs, Ms and Os for each theory. Theories were retroductively fine-tuned to guide their consolidation in phase 3. This process included structured analysis workshops, capacity-building webinars on RE and continuous formal (monthly teleconferences) and informal (email/Skype) exchange within and between the two collaborating teams. The outputs from this phase were eight theories in which we articulated causality through ‘in the context of.if.when.’ propositions. Each proposition acted as magnifying glasses that both augmented and focused our knowledge. A distinctive feature of this phase was our increased in-depth command of the literature, which became more focused on each PT. These eight theories are available elsewhere (Ebenso *et al.*, 2019, see supplementary file) and are summarized in Box 2, with refined security and trust PTs and their narrative causality propositions included in Figures 3 and 4 later.

Security was a clear first theme identified inductively during the workshop in February 2017 (Box 1), and deductive reasoning was then used to put this theory to the test through iterative engagements with the literature, data and discussions within research team. As shown in Figure 2, it subsequently became a separate supply-side PT S3 (health workers feeling secure to provide health care) and a component of the demand-side PT D1 (feeling safe as part of being supported to utilize MCH services).

Trust, however, did not feature prominently at this stage as a distinct PT but cut across some of the initial 15 themes shown in Box 1, such as those associated with relationship building (theme 10) and different types of financial and non-monetary incentives to improve staff performance in providing MCH services (themes 2–9). During this phase, trust became a component of several PTs shown in Figure 2, specifically S4 (implications of withdrawal of support on the provision of health care) and D4 (sustainability of trust in the utilization of health care) and also cut across PTs on motivation to provide and use MCH care (S2, D2 and D1).

Following on from the magnifying glass metaphor, during this phase, specific literatures acted as lenses that framed our analysis. On reflection, our theoretical engagements with the literature were still too close to the level of the empirical with lesser engagements with substantive social science theories, which are necessary to fully establish causality in REs (Chen, 1989; Rogers and Weiss, 2007) and inform the middle-range theories (Merton, 1968; Pawson and Tilley, 1997).

### Phase 3. Into the social laboratory: theory testing, refinement and consolidation

During phase 3, we iteratively tested, refined and consolidated the PTs. At a meeting in September 2017, we developed a detailed fieldwork work plan, which included a wider range of methods—all summarized along with detailed guidance in our methodology handbook. During phase 1 and parts of phase 2, separate fieldwork staff collected and transcribed data under the guidance of more senior core project team who then subsequently analysed the data. Each SURE-P/MCH cluster evaluated was assigned eight fieldworkers. Subsequently, in phases 2 and 3, both data collection and analysis were conducted by the core researchers at the University of Nigeria comprising 10 members. All these raised the importance of having a clear and dynamic (modified for each phase) handbook with step-down capacity-building workshops in the University of Nigeria.

While we used methods that were similar to previous phases, the data collection instruments were updated for the testing of PTs. For example, interview guides were structured around causal links within PTs rather than separate Cs, Ms and Os (Manzano, 2016). A previously conducted facility exit survey did not capture views from those who did not seek health care from health facilities, and to fully understand trust and other PTs, we conducted a household survey.

The scale of the data collection during phase 3 was smaller (39 IDIs and 4 Focus Group Discussions (FGDs) as compared with 96 IDIs during previous phases). However, depth of our engagements with the results was greater as we refined the PTs. At a meeting in February 2019, we extensively discussed, peer-reviewed and further refined the PTs. We also deepened our understanding through visualizing causal links within each PT (see Figures 3 and 4 for security and trust, respectively). These figures, in addition to articulating refined narrative causal proposition for each PT, also show which specific elements of macro and institutional contexts (numbered Cs) trigger specific reasoning or resources/intervention aspects of mechanisms (Dalkin *et al.*, 2015) (numbered Ms) and which outcomes (numbered Os) are intended, unintended and observed. Such visualizations helped the team to further refine and consolidate each PT.

Trust and security became distinct PTs and were consequently tested, refined and consolidated retroductively by synthesizing insights from three sources (literature on established social science theories, SURE-P/MCH programme design and mixed-methods data analysis) in what we call the ‘social laboratory’. The role of substantive social science theories in disentangling the specific mechanisms was operationalized. After substantive social science theories were identified, we did not collect further primary data but applied these new theoretical perspectives to our already rich datasets.

For security, we drew upon a complementary body of interdisciplinary literature on theories of fear of crime and gender which determine perceived security by health workers and patients (Hough and Mayhew, 1983; Bennett, 1990). This literature related fear of crime to structural factors like physical layouts of buildings (Dammert and Malone, 2003), political, socio-economic issues, insecurities like poverty and place of residence (Stanko, 1987; Pain, 2001). Although security was not explicitly articulated in the design of the SURE-P/MCH, some health facilities with the support of local communities erected perimeter fences, improved lighting and employed night-time guards—which altogether contributed to reduced fear of crime from staff and patients and ultimately contributed to improved provision and uptake of MCH services.

Substantive theories used to approach causality in the trust PT were progressively selected to capture the complex structural relationships that are entangled across trust-related micro, meso and macro factors (Straten *et al.*, 2002) from the programme’s investment in deploying and training staff and conditional cash transfers. To understand trust, we used taxonomies of factors, which lead to trust such as safety, benevolent concerns, capability, predictability and communication (Gilson, 2003; Hurley, 2006). We also used a social capital theory (Szreter and Woolcock, 2004; De Silva and Harpham, 2007; Agampodi *et al.*, 2015) to interpret the sustainability of ‘residual’ trust by patients after the funding for SURE-P/MCH ceased, which we found from our quantitative data.

We faced three conceptual challenges: first, continuous toggling across the levels of abstraction (CMO configurations in testing PTs and broader middle-range theory). This involved balancing between comparing and contrasting our results with empirical findings from health systems literature at the level of CMOs and a more theoretical engagement with substantive theories at the level of middle-range theory. Second, reaching shared understanding on ‘what causes what’. This meant often moving Cs, Ms and sometimes Os around and resolving circular arguments while going beyond the conventional ‘everything leads to everything’ conclusions. Last, the decision as to which substantive social science theories to use in trust and security PTs. This was perhaps one of the most challenging aspect as there is no firm protocol for appropriateness of theories in realist studies, and which reflected different approaches we used for theorizing security (drawing on complementary body of literature) and trust (progressive selection of theories). Ultimately, the iterative nature of dipping in and out of theories and the data helped us to continuously validate our theorization including choice of appropriate theories and empirical backing of PTs.

## Discussion

Neither security nor trust was explicitly articulated in the design of the SURE-P/MCH programme but emerged from data and substantive social science theories. Security appeared a more tangible and context-specific PT, so emerged early. However, security literature often focuses on global/national security threats, and fear of crime is used as a proxy for security itself. Furthermore, tangibility does not always ensure attention in policy design. During our feedback workshop in January 2020, Nigerian policymakers reflected that, since security is such a mundane and routinely evident issue, they had never linked it with provision or uptake of health care. This was mirrored by the scarce literature on the security of health facilities. Conversely, trust was a softer and less tangible concept so emerged later in the project. Trust cut across and was reflected in multiple (often opposing) PTs, perhaps reflecting a larger though complex body of literature on the topic and argument that it applies to all national health systems (Gilson, 2003).

A significant distinction between the development and testing of the two PTs was the data used. The dataset for the security PT was exclusively qualitative. However, the trust PT used both quantitative (survey and facility data on service utilization, which highlight residual trust) and qualitative interview data. The tangibility of supporting evidence on security (such as knowledge of which facilities had security measures such as perimeter fences, security guards) meant that we did not need to collect quantitative data. Given that the trust PT addressed a softer, less tangible concept, we needed insights from multiple quantitative and qualitative methods to sufficiently disentangle this phenomenon.

Our processes of theory tracing followed the established guidance on REs (Wong *et al.*, 2016a; Emmel *et al.*, 2018). The long-term nature of our study allowed adequate engagements throughout the project phases with stages of theory gleaning, developing, testing, refining and sourcing relevant nuggets of evidence for our PTs (Pawson and Tilley, 1997; Pawson and Manzano-Santaella, 2012; Manzano, 2016), thus highlighting the need for substantial investment of time and expertise required in REs. While we did not conduct follow-up data collection based on substantive social theories, we identified and articulated irregularities within grouped PTs, e.g. through consolidating elements of trust into a distinct PT (Gilmore *et al.*, 2019).

Collaborative research typically builds on complementary expertise of different research team members across the partner organizations (Denis and Lomas, 2003; Shulha and Wilson, 2003). Our experience also echoes the argument that interdisciplinary expertise—in our case, combination of social sciences, health economics, health systems and policy research—is useful for developing context-sensitive theories (Emmel, 2019). However, collaborative studies also require a shared understanding of the underlying ontologies and epistemologies. This is particularly important for partners who work across countries and often face resource constraints and imbalanced power relations (Abimbola, 2019; Gilmore, 2019). To reach such an understanding we had teleconferences, webinars, face-to-face capacity-building sessions followed by step-down capacity-building workshops. Adequate resources and appropriate processes to ensure rigorous analytical engagements across the organizations are therefore essential within collaborative REs.

Our overarching argument, and a lesson, is that REs are important, though time-consuming, approaches to generating practical implications for policy and practice (Kwamie, 2016; Van Belle *et al.*, 2017). As such, REs reflect an applied nature of health policy and systems research (HPSR). Our security and trust PTs exemplify the applied nature of REs within the HPSR through being context specific and policy relevant. In the ever-changing LMICs policy context, REs offer a flexible approach (Wong *et al.*, 2016b), e.g. through opportunities to continuously reshape PTs to address changing priorities and decision-makers’ interests. This was evident after the SURE-P/MCH was ceased, when multiple advocacy and lobbying initiatives to maintain political prioritization of MCH informed our PTs on advocacy. A key question, however, is how quality REs can be assured within the contexts of rapidly changing priorities and constrained resources in LMICs.

At the same time, appropriate levels of continuous theorization are essential for good-quality REs (Greenhalgh *et al.*, 2017b; Marchal *et al.*, 2018; Gilmore, 2019). The increasingly accepted ladder of theoretical abstraction in realist research, which starts with individual findings and then progresses to PTs and associated CMO configurations and eventually generalizable middle-range theories (Pawson and Tilley, 1997; Greenhalgh *et al.*, 2017b; Marchal *et al.*, 2018), offers a useful framework to help disentangle levels of theorization at different stages of realist research. However, multiple models of PTs and CMOs (Funnell and Rogers, 2011; Adams *et al.*, 2016; CDI, 2016), coupled with expectations that middle-range theories still being close to observed patterns in the data (Merton, 1967; Greenhalgh *et al.*, 2017b), can raise challenges in the consistent application of appropriate levels of theorization for realist researchers. Some ‘toggling’ between different levels of abstraction is inevitable, as we also faced in our study, and researchers need to be self-aware of such ascents and descents and be able to articulate their theories at the ‘right’ level to balance theoretical generalizability and application to policy and practice.

Our overall argument is supported by three lessons learned. First, theories in REs cover the continuum of middle-range theories—PTs—CMO configurations (Marchal *et al.*, 2018). It is important to recognize these different levels of abstraction including the interdependencies and overlaps between them and ensure that PTs are ‘pitched at the right level’ to maintain methodological rigour alongside the policy relevance.

Second, theory gleaning, development, testing, refinement and consolidation require that different nuggets of evidence (documented programme design and implementation, theoretical and empirical literature, primary data) converge timely and usefully (Pawson and Tilley, 1997). This is always a challenge in REs, but the context of LMICs increases such challenge where due to resource constraints, programme design and implementation are often insufficiently documented and researchers face difficulties accessing relevant literature.

Third, the retroductive approach in REs requires iterative and flexible data collection and analysis against the literature, meaning that different theories need to follow their own (often different) processes of gleaning, development and testing. Social scientists can be useful ‘theory brokers’ when proceeding to the ‘social laboratory’ stage. Collaborative studies involving partners from multiple countries face a challenge of a ‘parachuting’ approach leading to fragmented engagements—and instead need to maintain the continuity, coherence and shared understanding of the analytical processes. Our long-term study allowed for in-depth engagements and studying the sustainability of programme outcomes such as trust. However, as we faced in our project too, it is important to maintain the momentum and focus within longitudinal studies.

## Conclusions

In this article, we documented experiences of gleaning, developing, testing, refining and consolidating two PTs (security and trust) from a longitudinal RE of a national health programme in Nigeria. Our overarching argument, and a lesson, is that REs are important, though time-consuming, approaches to generating practical implications for policy and practice and that each theory development process is always complex, unique and distinctive. Three practice lessons are proposed for researchers and practitioners who are interested in understanding specific theory-tracing processes and a wider utility of REs. First, PTs need to be at the right level of abstraction to maintain methodological rigour alongside the policy relevance. Second, sourcing relevant nuggets of evidence is essential for theory tracing and is often a challenge in many resource-constrained settings. Third, collaborative REs require social science expertise and continuous theoretical engagements maintain the continuity, coherence and shared understanding of the analytical processes.

**Box 1 Themes from a workshop in February 2017**

1. Security
2. Staff Accommodation
3. Availability of Adequate Number and Skill Mix of Health Workers
4. Availability of Drugs and Consumables
5. Availability of Equipment
6. Availability of Health Facility Infrastructure (Upgrade/Borehole/Solar Power)
7. Training
8. Supervision
9. Salaries and Incentives (Demand and Supply-Side Incentives)
10. Relationship Building

  a.  Health Worker/Health Worker Relationship
  b.  Health Worker/Community
  c.  Health Worker/Service User
11.  Transportation for Community Mobilization
12.  Referrals
13.  Intersection of Programme Components
14.  Political Commitment
15.  Others

**Box 2 Summary of eight PTs from phase 2**

S1: Prioritization of MCH. In the context of poor health outcomes, interest from policymakers and politicians in MCH care, combined with advocacy and lobbying from key policy actors to prioritize MCH (C), is likely to help generate and maintain political and economic commitment across all tiers of government manifesting as a culture of ownership of MCH programmes (M), eventually leading to timely release of counterpart funds and availability of other resources (e.g. human, supplies), ultimately contributing to sustained implementation of and access to MCH services for vulnerable groups (O).

S2: Improved staff motivation. In the context of staff shortages and lack of material resources, if adequate numbers and mix of skilled health workers are recruited, deployed to health facilities (that have security men, comfortable accommodation, regular electricity and water supply and transportation for emergency referrals) and if health workers receive adequate equipment, supplies and consumables for their work and are regularly trained, supervised and rewarded for good performance (C), then health staff will feel motivated (i.e. appreciated and happy) to increase and maintain their performance (M), which is likely to lead to increased provision and utilization of quality MCH services, ultimately contributing to improved service and health outcomes (O).

S3: Health workers feeling safer to work. In the context where health facilities or communities ensure employment of security men, erection of perimeter fences and availability of accommodation for health workers in the facility premises (C), then health workers are likely to feel safer and therefore willing to work (i.e. provide MCH services) during night hours (M), thus ensuring the provision of round the clock MCH services, and improved access to MCH services (O)

S4: Implications of withdrawal of support. In a context where basic support to health facilities (e.g. staff salaries, electricity, equipment and supplies) is dependent on project funding, a sudden withdrawal of political and financial support to previously funded MCH programme will limit the availability of human and material resources (C), making health workers feel unappreciated and unsupported (M), resulting in low morale and distrust among health workers and reduced performance, which can ultimately constrain the sustained provision of MCH services (O).

D1: Supporting women to access health care. In the context of pregnant women’s inability to pay for transportation to health facilities, or for medicines and MCH services, if Ward Development Committees (WDCs) are mobilized and trained and pregnant women are provided conditional cash transfer (CCT) and if village health workers (VHWs) encourage and support women to attend MCH services (C), then pregnant women will feel safer and confident to regularly attend health facilities (M), thus leading to increased and sustained utilization of health facility-based MCH services (such as ANC, deliveries and postnatal care), and ultimately to better MCH service outcomes (O).

D2: Motivated and better performing VHWs. In the context where pregnant women are financially incentivised to access MCH care, if VHWs are provided with CCT and means of transportation to enable them mobilize communities and support women to reach hospitals (C), these VHWs will feel more recognized by communities and motivated to encourage and accompany pregnant women to facilities for MCH services (M), thus contributing towards increased and sustained utilization of MCH services by the pregnant women (O).

D3: Distrust due to withdrawal of incentives. In the context of on-going targeted programme to improve access to MCH services to vulnerable pregnant women from remote rural areas, the sudden withdrawal of monetary and non-monetary incentives to support pregnant women to attend the continuum of MCH care (C) will help generate distrust from these women of health workers and wider system and demotivate pregnant women from attending health facilities (M), eventually leading to the reduced utilization of available facility-based MCH services (O).

D4: Sustainability of trust in the health system. In the context of improved staff attitude, upgraded health facilities and functioning WDCs achieved during implementation of the SURE-P programme, pregnant women who receive sustained financial and non-financial incentives to use MCH services (C) are likely to develop and maintain a sense of improved trust (including confidence and satisfaction) with health facilities and staff (M), ultimately leading to improved likelihood of repeated and regular utilization of MCH services from these health facilities (O).
